# Deployment of dentists in COVID-19 case investigation and contact tracing: An example from Turkey

**DOI:** 10.3205/dgkh000453

**Published:** 2023-11-29

**Authors:** Ozlem Ozkan, Neslihan Sevim, Zeliha Ocek

**Affiliations:** 1 Department of Health, Kocaeli Academy for Solidarity, Kocaeli, Turkey; 2 Dental Clinic, Ankara, Turkey; 3 Institute for Medical Information Processing, Biometry, and Epidemiology (IBE), Chair for Public Health and Health Services Research, Medical Faculty, LMU Munich, Munich, Germany; 4 Pettenkofer School of Public Health, Munich, Germany

**Keywords:** COVID-19, case investigation, contact tracing, dentist, pandemic

## Abstract

**Aim:**

In Turkey, dentists working in public dental care centers were deployed in COVID-19 case investigation and contact tracing (CICT) teams during the pandemic. This study aims to explore the experiences of the dentists assigned to teams undertaking COVID-19 CICT practices to determine how healthcare workers should be supported when working in pandemic response and other crises.

**Material and method:**

The sample of this qualitative, phenomenological study consisted of thirty four public dentists assigned to COVID-19 CICT practices in four metropolitan areas of Turkey. Data were collected through semi-structured in-depth interviews that were conducted online in August and September 2020. The data were analyzed using thematic content analysis.

**Results:**

Six themes were revealed: preparation for CICT, basic requirements, work relations, working conditions, being a dentist assigned to CICT and COVID-19 pandemic management. The dentists complained that they were not appropriately assigned to CICT, as they lacked the preparations and sufficient training. They had to acquire personal protective equipment and other basic needs at their own expense. The working conditions were severe, and they had negative relations at work. The State and the Ministry of Health were criticized for inadequate implementation of institutional measures for COVID-19 pandemic management.

**Conclusions:**

The study showed that dentists were motivated to participate in the management of pandemics and similar crisis situations, but in a negative work environment – where they were deployed without adequate training, preparation, and ensuring their basic needs and requirements were met – they lost this motivation, and experienced stress and feelings of inadequacy.

## Zusammenfassung

**Zielsetzung:**

In der Türkei wurden Zahnärzte, die in öffentlichen zahnärztlichen Versorgungszentren arbeiten, während der Pandemie in COVID-19-Falluntersuchungs- und Kontaktverfolgungsteams (CICT) eingesetzt. Ziel dieser Studie ist es, die Erfahrungen der Zahnärzte zu untersuchen, die in COVID-19-CICT-Teams eingesetzt wurden, um herauszufinden, wie das Gesundheitspersonal bei der Pandemiebekämpfung und in anderen Krisen unterstützt werden sollte.

**Material und Methode:**

In die Stichprobe der qualitativen, phänomenologischen Studie wurden 34 im öffentlichen Dienst beschäftigte Zahnärzte, die COVID-19 CICT-Praxen in vier türkischen Großstädten zugeordnet waren, einbezogen. Die Daten wurden durch halbstrukturierte Tiefeninterviews erhoben, die im August und September 2020 online durchgeführt wurden. Die Daten wurden mithilfe der thematischen Inhaltsanalyse analysiert.

**Ergebnisse:**

Es wurden sechs Themen identifiziert: Vorbereitung auf das CICT, Grundvoraussetzungen, Arbeitsbeziehungen, Arbeitsbedingungen, Einsatz als Zahnarzt im CICT und COVID-19-Pandemiemanagement. Die Zahnärzte beklagten sich darüber, dass es ihnen nicht leicht fiel, im CICT eingesetzt zu werden, da ihnen die Vorbereitungen und eine ausreichende Ausbildung fehlten. Sie mussten auf eigene Kosten Geld für persönliche Schutzausrüstung und andere Grundbedürfnisse ausgeben. Die Arbeitsbedingungen waren hart, und sie hatten schlechte Arbeitsbedingungen. Der Staat und das Gesundheitsministerium wurden für die unzureichende Umsetzung der institutionellen Maßnahmen zur Bewältigung der COVID-19-Pandemie kritisiert.

**Schlussfolgerungen:**

Die Studie hat gezeigt, dass Zahnärzte motiviert sind, sich an der Bewältigung von Pandemien und ähnlichen Krisensituationen zu beteiligen, aber in einem negativen Arbeitsumfeld, in dem sie ohne angemessene Ausbildung und Vorbereitung eingesetzt und ihre grundlegenden Bedürfnisse und Anforderungen nicht erfüllt werden, verlieren sie diese Motivation und erleben Stress und das Gefühl der Wertlosigkeit.

## Introduction

Although it is still prevalent and has affected all areas of life since 2020, the COVID-19 pandemic has been controlled primarily thanks to measures aimed at the source: the transmission route as well as the host. The main requirement for an effective implementation of these measures is the existence of a strong surveillance system [1], [2], [3], [4]. During the COVID-19 pandemic, great differences became apparent between countries with respect to the type of surveillance systems as well as case investigation and contact tracing. Such variations between countries also emerged in terms of the composition of the healthcare teams performing COVID-19 surveillance [5], [6]. 

### International examples for the inclusion of dentists in the surveillance of COVID-19

Although not very common, there are examples of countries where dentists were part of these teams. For example, in Singapore, dentists worked both in collecting nasal swab for testing and providing consultation [7]. Also, in Ghana, dentists worked in contract tracing teams [8]. In Turkey, dentists working for the Ministry of Health (MoH) were assigned to case investigation and contact tracing (CICT) [9]. Almost all healthcare workers (HCWs) assigned to COVID-19-surveillance have encountered a job description quite different from their usual responsibilities, and have been significantly affected by challenging working conditions [4], [5], [10], [11]. 

### Public dentists in COVID-19 CICT in Turkey

Globally, Turkey ranked 71^st^ (173,621) in terms of the number of confirmed COVID-19 virus cases per one million population on 06.04.2022 [12]. However, since the MoH (Turkey) reported only the exact number of cases and deaths in which SARS-CoV-2 was detected by molecular methods [9], the official statistics could be regarded as the tip of the iceberg.

CICT practice for any patient diagnosed with COVID-19 officially started on 25.03.2020 in Turkey by the establishment of local CICT teams in the district directorates of health in each province. Within these three-person teams, the main responsibility was assigned to the dentists working in dental clinics of the MoH. Alongside nurses and midwives, non-HCWs have also been assigned to work in CICT teams. Of the 4,600 CICT teams as of March 2020, approximately 25% were in Istanbul, 10% were in Ankara and less than 1% were in Izmir. The total number of HCWs working in the teams was reported to be 58,500 in June 2020 [13], [14]. Based on the information that 11,588 dentists were working at dental clinics of the MoH by 2020 and about 85% of those dentists were assigned to CICT practices in March–April 2020 [10], the number of dentists in CICT practices could be estimated to be about 10,000. 

CICT practice in Turkey is initiated through an online information system, where the registration of those with a positive COVID-19 test result, including household and employment details, is initially transmitted to the database of district directorates of health and then to the mobile application called Filiation and Isolation Tracking System (FITS), primarily used by CICT team members through their smartphones. CICT teams send a text of information/consent both to the positively diagnosed person and associated contacts and conduct visits to the household and workplace. During the visits, the teams take nasal swabs, bring in medicine for patients who are not hospitalized but having symptoms that require treatment, provide counselling about drug use, the isolation process and COVID-19, as well as complete registration procedures [13], [14] , [15]. It has been reported that CICT teams monitored an average of three contacts per case and worked 10 hours a day [15].

### Aim of the study

This study aims to explore the experiences of the public dentists assigned to teams undertaking COVID-19 CICT practices in four metropolitan areas of Turkey, reveal the challenges facing the HCWs in primary health services during the pandemic, and identify the ways in which HCWs with no prior training or experience in extraordinary health situations – including pandemics – could be supported. Identifying the barriers and facilitators faced by HCWs with backgrounds outside of primary care during COVID-19 surveillance will provide information about the training and support mechanisms that should be offered to healthcare professionals during the current pandemic and similar health crises.

## Material and method

The study was designed as qualitative research with a phenomenological approach. In this regard, we aimed to explore the CICT practices as described by the dentists in terms of both what was experienced and how it was experienced [16]. The study included dentists at the dental clinics of the MoH assigned to teams undertaking COVID-19 CICT practices in Turkey. The research sample was restricted to four metropolises (Istanbul, Ankara, Izmir and Diyarbakir) located in different regions of the country, where CICT practices were undertaken more intensely. In Turkey, 17% of 11,588 public dentists work at the dental clinics of the MoH in Istanbul, 13% in Ankara, 6% in Izmir, and 2% in Diyarbakir. Of these, the number of dentists assigned to CICT in 2020 was 1,250 in Istanbul, 500 in Ankara, 200 in Izmir and 140 in Diyarbakir [17]. The research sample was selected using a snowball technique and dentists working in CICT for least one week were included. Existing communication channels among dentists facilitated the exploration of a variety of experiences associated with CICT practices undertaken in different places, such as prisons or factories. The recruitment of the participants continued as long as new information was identified. Ultimately, the research sample consisted of thirty-four dentists (Istanbul=11; Ankara=9; Izmir=8; Diyarbakir=6). 

The data were collected through semi-structured in-depth interviews. The questionnaire was prepared based on a preparatory group interview held with two dentists and one public health institution manager assigned to CICT. The validity and comprehensibility were tested through pilot interviews held with three dentists who were excluded from the sampling population. The final form was composed of nine open-ended questions on the assignment process to CICT, preparations before the assignment, the daily routine of CICT teams, the notion of being a dentist in CICT, the challenges and motivation during CICT assignment. Participants were also asked about their socio-demographic features (age, gender, marital status, number of children if any, and household composition), experiences at work, and presence of chronic disease. Due to the lockdown and the 24-hour work cycle, all interviews were conducted online through Zoom between 11.08 and 28.10.2020. The interviews were recorded with the oral consent of the participants and lasted about one hour. 

The research was conducted under the approval given by the MoH General Directorate of Health Services and with approval from the Ege University Medical Research Ethics Committee (20-11.1T/35). Consent forms were signed by the participants and their oral consent was sought for recording the interviews. 

All transcripts were read by the authors immediately after each interview to identify an initial open set of codes and to outline notes for any striking issues not covered by the codes. Once this was completed separately by the authors, a common list of categories under each theme was identified collectively. After two of the authors coded all the transcripts according to this list, a second round of coding was conducted to fine-tune the codes to omit the unrelated ones and to add new ones. Then, the categories were structured along the conceptual framework where direct quotes from the participants were associated with each category. Finally, the analysis was completed by a validation check for the consistency across categories, codes, and associated quotes.

## Results

The study group consisted of thirty four dentists, more than half of whom were in 39–49 age group, female, married, with children and cohabiting with family members. One-third of the participants reported having a chronic illness. Three participants, two of whom were female, reported that they were infected by COVID-19 during their CICT assignments. The analysis resulted in identification of six themes and 21 categories (Attachment 1). 

### 1. Preparation for CICT 

#### Assignment 

Most of the participants (n=22) mentioned that their assigments were improperly organized. They were assigned without prior notification, and some were informed indirectly by their colleagues or by spoken declaration of their manager, via a whatsapp message or a phone call. Also, the assignment period was not specified for many participants and they went on mission either immediately after the notification or within two days’ time. After their initial assignment period, some participants were obliged to extend their assignment twice or three times. Dentists who had a chronic illness complained about their managers’ indifference to their health status while organizing their assignments. In contrast, some positive evaluations were given, such as the limited duration of the assignment, the presence of an official assignment letter, and the fact that certain conditions were considered, such as years of seniority or volunteerism.

#### Training

Twenty-six participants did not receive any training during their assignment, and seven participants had received insufficient training. The training provided lasted at most three hours, during which the participants received reading materials or training videos. In some cases, more experienced dentists were requested to provide peer training. The participants did not have time to read or work on the training materials provided. Only six dentists expressed their own effort to receive information by several means, such as reading the scientific literature, consulting family members who are doctors, or asking their own colleagues. 

### 2. Basic needs

#### Personel protective equipment (PPE)

All participants mentioned the quality and availability of PPE and especially the difficulties associated with both working in jumpsuits and donning and doffing them. Many of them complained about insufficient quantities of PPE and low level of quality. Women criticised the sizes of PPE that were designed for men. The participants had to repeatedly wear the same mask, gloves, and jumpsuits due to the lack of PPE. Around half of them referred to the difficulties of donning and doffing the jumpsuit. In this regard, participants had to use building entrances or the vehicles of the CICT team to change into or out of their jumpsuits. The jumpsuits were regarded as highly uncomfortable in hot weather or in cases where the building had many stairs to climb. As a result, many participants stopped using jumpsuits. In contrast, eighteen participants did not have any problems with the quantitative sufficiency of PPE due to donations, self-provisioning, or provision by the health directorate, thanks to pressure from the trade union as well as the chamber of dentists in later periods of the pandemic. However, the problems associated with the quality of PPE were mentioned as having persisted throughout the period of CICT assignment. 

#### COVID-19 testing

Half of the study group had problems related to access to tests, while six participants declared the opposite. Those unable to access the tests complained about the restrictions arising from the prerequisite that no tests were to be conducted unless symptoms were present. Two participants explained that tests became accessible thanks to the pressure of the dentists’ trade union and the chamber of dentists. 

#### Mobile communication

Mobile communication was the second most cited category among necessary prerequisites for CICT work (n=26). This category covered both phone calls and internet connection for FITS. The participants pointed out several problems, such as the use of their own mobile phone to call patients, calls from patients and their contacts at any time of day, low battery levels, inability to reach out to patients. Another issue was the slow pace of work on FITS and frequent disruptions in the system. The participants had to use their own (paid) internet connections for FITS and navigation, and experienced frequent interruptions in the connection in the rural areas visited. Only four participants had no difficulty with mobile communication, either because the smart phones were provided by the district health directorate, their phone numbers were not identifiable by the patient called, or there were sub-teams responsible for calling patients and a free internet connection package was provided.

#### Personal needs

Lunch and lunch break, drinking water, toilet, time and place for a rest break, and personal childcare needs were mentioned. Only five respondents had positive experiences, such as the allocation of a rest stop, and lunch provided by the provincial health directorate or the municipality. 

#### Security

Violence, traffic accidents, unsafe neighborhoods visited etc. were mentioned by twenty-five participants. Eight dentists reported violence against their colleagues, while seven had faced the risk of violence themselves. Five participants reported that they or their colleagues had been in traffic accidents. Participants (n=9) who had no experience of violence attributed this to the fact that they did not insist on any procedure, such as collecting nasal swabs, in the presence of a negative reaction. 

#### Transportation

Two out of every three participants experienced transportation-related problems during their daily commute and CICT assignments. Irregular working hours, working late, and lack of corporate shuttles or personal vehicles were transportation-related problems. Other problems included inaccurate addresses, long distances, poor quality of roads in rural areas and all other driving-related issues. Some of the dentists drove themselves and others had a chauffeur (driver of a municipal/directorate vehicle). Chauffeurs failed to use navigation apps, drove unsafely, did not wear masks, and were frequently changed by district health directorates. On the other hand, eleven participants had no problems because they had their own vehicle or could use a colleague’s vehicle, the driver knew the roads, the distance between home and work was relatively short, and the municipality provided a shuttle service for their transportation. The fact that dentists met their basic needs out of their own pockets was an important point of criticism. 

### 3. Work relations

#### Relations with the public

All participants complained about the negative attitudes of the public towards them. People objected to having nasal swabs, CICT teams were mistaken for thieves and prevented from entering buildings, even reported to the police. Some women said they would only allow female HCWs to take samples from them. Some patients tried to hide from their neighbors the fact that their homes were being visited by CICT teams. Participants also complained about frequent phone calls from patients at all hours of the day. In contrast, twenty-one participants emphasized the positive attitude of the public, who sincerely appreciated their efforts.

#### Relations with managers

Negative statements about the managers at the health directorate were reported by twenty-eight participants, such as mismanagement, inability to organize the work schedule of the teams, lack of improvements in working conditions, high turnovers among team members, inability to maintain coordination among the teams as well as between the teams and the family physicians, inability to communicate with dentists’ managers, applying pressure, threats, mobbing etc. and unequal treatment of HCWs. The participants also complained about the neglect of their demands for PPE, health status, personal needs, leave of absence, provision of support staff etc. The managers were identified as having assigned dentists to all the duties associated with CICT practices. In this regard, such negative experiences about the relations were identified as the most challenging issue. On the other hand, participants having identified positive relations with the district health directorate (n=23) indicated that managers had been working assiduously, taking the dentists’ demands into consideration, and had been able to resolve the once problematic issues of provision of lunch, venue for the rest break and PPE. In fact, in some cases, managers were described as co-working with the health teams in CICT. 

#### Relations with CICT team

The participants referred to negative experiences with the CICT teams, such as inadequate number of health staff in the team, incompatible work with the chauffeurs, high turnover rates for the team members, and incompetence of team members assigned to CICT missions from professions other than HCWs, i.e. teachers, officials, caretakers etc. Fifteen participants mentioned that the composition of the team remained inadequate to meet the needs on mission, and emphasized the overall inadequacy of the HCWs in terms of the quantity as well as the variety of expertise. In relation to the issues with chauffeurs, sixteen participants indicated a lack of safe driving, knowledge about the roads, ability to use navigation, compliance with hygienic measures, proper communication manners, and the intention to synchronize with the work duration. On the other hand, nineteen participants mentioned that the team did not change throughout the assignment, and that they worked in harmony with the team members who demonstrated dedication to the job. 

### 4. Working conditions

Thirteen dentists reported positive experiences concerning working conditions in the later stages of the CICT, such as the regulations of working hours, leave of absence, and rest break. Contrarily, thirty one dentists complained about working conditions that were excessive, involved long hours, were irregular, intensive, and uninterrupted, without rest.

### 5. Being a dentist assigned to CICT

#### Family life

Complaints about the impacts of the assignments on family relations were raised by twenty four participants. Dentists had to send their families away to live with relatives living in other provinces and were unable to visit elderly or chronically ill relatives, as they were worried about transmission of COVID-19. Moreover, dentists faced the pressure of their families to leave the CICT. One female dentist had even been denied an official leave of absence when her father passed away. In contrast, four dentists did not experience problems because their houses were were large enough to enable distancing from other family members, they lived alone, their children were adults, and they did not tell their families that they were assigned to the CICT.

#### Volunteering

Twenty respondents were not willing to work for CICT, despite having initially volunteered to work at CICT. Eleven respondents never volunteered and fifteen continued to volunteer to work at CICT. Those who had initially volunteered to work at CICT explained that they did so because CICT was more advantageous than their previous workplaces, they wanted to contribute to pandemic management, assume social and professional responsibility, assist in mobilization, or they saw the pandemic as a “war” situation, and wanted to ease the burden on their colleagues and other HCWs. Six dentists found the flexible work in CICT more attractive. Almost all dentists emphasized that earning more money was not important in a decision to volunteer, but it could be important for their colleagues. Some also mentioned that their opinions towards volunteering had changed in due course. These changes were attributed to factors such as non-consensual tours of duty on their CICT assignments, tasks outside their job descriptions, ineffectiveness of CICT in controlling the pandemic and under-appreciation of their efforts. A significant group of dentists also mentioned the unfavorable working conditions and the impact of their unmet basic needs during the missions. Six dentists would not recommend CICT assignments to any of their colleagues.

Those who initially did not volunteer for CICT said that there was uncertainty, that the tasks related to their profession were not being performed, and that they did not want to be exposed to the risk of COVID-19. Those still volunteering referred to unfavorable working conditions at their former dental clinics and the continuation of the mobilization conditions for the fight against the pandemic. Five dentists became accustomed to CICT and four dentists emphasized the need to serve the nation regardless of the prevailing conditions.

#### Job description and competence

All participants referred to nasal swab collection as a task within their job description and competence. However, twenty-nine respondents indicated that their work at CICT fell outside the boundaries of their own job description and areas of competence; moreover, the content of their work at CICT had been continually changing. All participants stated that taking responsibility for deciding on the use of the drug “Plaquenile” and delivering this drug to the patient at home was definitely not part of their job description and competencies. In this regard, some dentists even identified themselves as a “delivery person”. Other tasks outside the job description and competencies included writing medical reports for COVID-19 patients and other registration tasks. Four dentists complained about making home visits for the CICT and two dentists criticized inspecting weddings and shops. They even described themselves as "municipal police and joker staff" for doing so. On the other hand, seventeen participants viewed tasks in CICT as necessary and appropriate for their profession. Some participants also referred to the pandemic as a “war”, where all tasks would be fulfilled under mobilization regardless of their status as exceeding their job description. 

#### Payments

Almost all respondents complained that they had been unable to receive the payments they deserved, while performance-based payments had become irregular and low after the first three months. Some respondents even pointed to the inequalities among payments. 

#### Dentist from MoH perspective

Twenty-three dentists complained that dentists assigned to CICT practices had no value in the eyes of the MoH, were marginalized, isolated and not consulted about the CICT. In this regard, they expressed that the MoH treated them as a “reserve workforce, auxiliary HCWs and even step-children”. 

#### Emotions

Thirty-two dentists reported negative emotions related to CICT. They mentioned uncertainty, uneasiness, feeling helpless in relation to lack of access to COVID-19 tests, anxiety about infecting family and society, exclusion, dissatisfaction with work, alienation, unhappiness, loss of motivation and meaninglessness due to mismanagement of COVID-19 and blankness. Such negative emotions were expressed more by those who had a second or multiple assignments, and those with non-consensual extended tours of assignment to CICT. Some participants even started taking medication, using antidepressants and analgesics. In contrast, seventeen dentists expressed positive emotions, especially during the first assignment for CICT. They felt better because of having a chance participate in fighting against the pandemic, which has been regarded by many as a “war”. The reasons they gave for their positive emotions were their impression of being useful and helpful in the management of the pandemic, appreciation from society, and solidarity among their personal/kinship networks.

### 6. Management of the pandemic

#### State

Sixteen participants reported government failure to manage pandemic. The reasons were stated as lack of lockdown restrictions, neglect of necessary surveillance by provincial governorships and security forces, permits granted for social events and gatherings (including weddings), lack of economic support for those unable to work due to COVID-19, either as the infected case or contact associated with the case, and early introduction of normalization with the removal of all restrictions. 

#### Ministry of Health (MoH)

The MoH was regarded by thirty-two participants as unable to manage the pandemic, while three participants expressed that the MoH had been showing the utmost effort. The complaints were that the MoH failed to coordinate the field missions, district-level health directorates and family physicians, the infrastructure of CICT was not appropriate, the assignments to CICTs were either not issue to other HCWs, including family physicians, or given to those who were not HCWs, and that CICT remained insufficient due to the lack of primary health services. Moreover, the dentists severely criticized the fact that upon completion of their assignment at the CICT, they were immediately reassigned to their previous workplaces without necessary precautions being taken. The participants also referred to the differences between the practices of district health directorates, the competition existing among them, and the lack of any regulation to modify the working conditions and to improve personal rights at work.

The MoH was also criticized for the lack of official announcement of total number of infected cases as well as total number of deaths related to COVID-19 among HCWs and dentists, the neglect of COVID-19 as an occupational disease, and the unavailability of routine COVID-19 testing for the HCWs. 

#### Public

Twenty dentists criticized the public for not using masks or not using them correctly, and for going out when they were in quarantine or isolation. The existence of social activities such as weddings and the continuation of the work due to the lack of economic support mechanisms were among these criticisms. Such problems were referred to as aggravated due to lack of provision of necessary measures and premature easing of restrictions by the state. 

## Discussion

This research provided in-depth information on the experiences of dentists assigned to COVID-19 CICT practices in Turkey. It may be regarded as one of the very few studies conducted in Turkey and other countries. With the COVID-19 pandemic affecting the entire world, pandemic preparedness has become even more important. It is extremely important for countries to be prepared for such pandemics and similar crises, not only to prevent them and control their effects, but also to improve the well-being of HCWs and to activate mechanisms to cope with new and challenging conditions [18]. The emphasis given by the dentists in this study to the problems and associated coping strategies could be regarded as an indication of their unpreparedness for pandemic. The evidence from research on other countries’ experience also indicates similar findings, showing that healthcare systems and HCWs – including dentists – were not prepared for the COVID-19 pandemic [11], [19], [20], [21], [22].

The inappropriate assignment of dentists to CICT, disregard of their chronic diseases, their inexperience with primary health services, and a failure to provide necessary training constitute the set of potential barriers to their ability to develop coping strategies against the challenges facing the dentists in CICT. One of the important tools to overcome such barriers is the training in the CICT tasks. As a matter of fact, some evidence points out that the dentists and HCWs who received training prior to the pandemic were more prepared for the pandemic and demonstrated higher levels of awareness [18], [23], [24]. Moreover, such training was regarded as vital for the assignments of inexperienced staff to CICT; CICT staff were provided with all necessary training in some countries [4], [5], [11].

When faced with serious problems in meeting their basic needs during the CICT missions, such as lack of access to equipment including PPE, dentists demanded public or institutional provison. Given that they were in close contact with COVID-19 patients during indoor nasal swab collection, the number of asymptomatic cases were high, and vaccination had not been initiated by the time our research was conducted, the public provision of PPE and regular COVID-19 testing remained critical. However, similar to the findings in our research, the evidence points to the inadaquate quantity of PPE as well as nonstandard and inappropriate design of PPE, especially for women, as the most-cited PPE-related problem by the dentists and HCWs [21], [25], [26], [27], [28], [29]. Non-publicly funded PPE also puts dentists at a high risk for COVID-19 infection and would even lead to cross-infection [30]. This study’s finding that dentists were declared to be at high risk of COVID-19 due to above mentioned factors is also supported by similar evidence from research in other countries [24], [28], [31], [32]; ultimately and consequently the dentists did not volunteer to work in CICT. In addition, similar findings were reported on dentists’ experience of physical health problems, such as difficulties in breathing, hyperhidrosis, tachycardia, fatique and weight loss because of spending a longer time wearing masks and jumpsuits over the course of the missions [33], [34]. 

Despite the recommendation for extensive routine testing for HCWs, the policies for testing vary significantly among countries [35], [36]. In Turkey, similar to the practice in many countries, tests were implemented only in case of existing sypmtoms. In this regard, our research with dentists assigned to CICT revealed demands for regular public provision of testing and complaints about the ineffectiveness of the MoH activities in this regard. 

The relations of the dentists with their managers, with the health team, their colleagues and the public were substantially affected in this pandemic [28]. Our findings indicated that dentists experienced problems mostly with the people they served/general public, including violence or mobbing faced by the dentists or their team members, phone calls by the COVID-19 patients or relatives any time during the day, and negative attitudes of the people visited, as they felt stigmatized. The problems with the managers ranked second, while problems with the health team ranked the lowest of all. Such negative experiences of dentists with the public and managers resembled the experiences of dentists and HCWs in other countries [5], [19], [24], [28]. The evidence on negative experiences of the dentists with the public was an unanticipated result, because especially in the first year of the pandemic, HCWs were very much appreciated by the public in every country. Such negative experiences might be attributed to the lack of clarity surrounding the pandemic in its early phases, the accelered increase in the number of cases and the lack of measures taken against this trend. 

HCWs and dentists had to work under unfavorable conditions during the COVID-19 pandemic, e.g., heavy workloads, without proper rest breaks or even lunch breaks, and irregular and longer working hours work [24], [25], [28], [37], [38], [39]. Similar problems were also raised in a study on dentists involved in CICT [10], [40]. For this reason, the most frequently cited problems included lack of access to PPE, unfavorable working conditions and irregular/inadequate payment [19], [20], [23], [25], [28], [29], [30]. Actually, in terms of problems with working conditions, some of the dentists pointed out that they either volunteered to work in CICT or were willing to ask that their current assignment at CICT be prolonged, because of more severe working conditions in the insitution at which they normally worked. In this regard, more severe conditions at their prior workplace made their adjusment to the working conditions at CICT easier [10]. 

The present findings on the unfavorable work conditions and work relations, low level of payment, and extra expenditures related to the pandemic all had a negative impact on the dentists’ family life and perception of the profession. In line with evidence from other studies, the risk of infecting their own family members imposed an additional psychological burden on the dentists [10], [21], [30], [31], [32], [38]. As a response to this, the evidence from our research revealed that dentists assigned to CICT had to send their family members to a relative’s house or refrained from any physical contact in the same house, as also identified in another study [29]. The novel finding of our research is the imbalances and deteriorations in family life of those assigned to CICT due to their irregular and overburdened work schedules and the difficulties encountered when fulfiling the tasks associated with CICT. 

The dentists in our study complained about the frequent changes in the content of the tasks in CICT. Their views on these tasks could be categorized in three groups. The first group referred to the task of the nasal swab collection for COVID-19 testing as an integral part of their competence. The second group cited the tasks of decision making on the use of the medication Plaquenile, delivery of medication, and surveillance at wedding ceremonies and shop visits as being beyond their job description. The dentists who were aware of such unfavorable conditions had not volunteered for CICT assignments at the initial stages of the pandemic, and those who volunteered but had no prior knowledge on those had in due course withdrawn from voluntary participation. Unfortunately, there is no other available evidence to discuss these findings. The third group referred to the nature of the tasks in the CICT as those that had to be fulfilled under mobilization or “war” conditions related to the pandemic, despite being outside their job description or professional competence. Interestingly, these reasons were also mentioned as justification for those who volunteered for CICT. The portrayal of a pandemic as a war or a state of mobilization is simply a reflection of the populist discourse used by the political leaders, especially in countries like Turkey, Brazil or the USA, to reinforce the perception of the pandemic as an external threat [41].

The evidence from research on HCWs and dentists in CICT identified several psychological problems, such as occupational burnout, stress [10], anxiety, fear [29], [42] fatigue, sadness, pessimism, dissatisfaction, crying jags, disrupted sleep patterns, and loss of energy [40]. Similarly, the studies on dentists working in different institutions identified experiences of anxiety, uncertainty, depression, occupational burnout, despair and loss of control, sadness, fear, anger, depression [21], [23], [24], [26], [32], [39], stress, disrupted sleep patterns [24], [26], [28], [32] and psychological distress [32], [37], [38]. The dentists in this study experienced emotions including disappointment, as also mentioned by other research [19], uneasiness, exclusion, professional dissatisfaction, alienation, unhappiness, as well as meaninglessness and loss of motivation related to the inefficiency of all tasks undertaken during CICT missions to mitigate the pandemic. In line with the findings from other research, the under-appreciation of the dentists was associated with a lack of routine testing for them, problems in access to PPE and lack of interventions by the MoH to ameliorate the working conditions and uphold rights at work [23], [26], [28], [32], [42]. Also, similar to the results of other studies, fear, anxiety and uneasiness were associated with the risk of infecting the family members and the patients they work with [21], [26], [29], [37], [38]. Moreover, in line with the findings in one study [10], occupational burnout and stress were associated with the obligation to perform tasks that were outside their job descriptions, the frequent changes of these tasks, severe working conditions and unfavorable work relations. Similar to the evidence from one study, uncertainty was associated with the uncertainty about the duration of the pandemic as well as the ambiguity about both additional payment and the duration of the assignment [23]. In addition to those, the dentists mentioned that they experienced exclusion, under-appreciation and isolation, because of the non-fulfilment of the MoH interventions for improving the existing working conditions and that they were viewed as “reserve workforce, auxiliary HCWs and even step-children”. In other research about actions not taken by government and health officials to protect their profession, dentists also expressed their disappointment [38]. All these negative emotions indicated that dentists were in need of psycholocial support [21], [29]. As such, the emotions not only affected the mental health and decision-making ability of the dentists, but also diminished the quality of the services provided under CICT [37].

Dentists identified the state the MoH in particular – as having inadequately undertaken institutional measures in the pandemic management. The common complaints raised against these institutions included ignorance of the work undertaken at CICT missions, lack of recognition of COVID-19 as an occupational disease, and lack of information on the number of COVID-19 cases and COVID-19 related deaths among HCWs. Their complaints about ignorance were similar to the findings in another study [19]. The efficient management of pandemics by the state would not only contribute to making HCWs feel safe but also enhance the quality of the provision of public services. In a similar vein, dentists in India pointed out that they would feel safer if the governments passed further legislation regulating their professional commitments [43]. The views of the dentists on the mismanagement of the COVID-19 pandemic by the public could have negatively affected the relationships with other people encountered on CICT missions, as also indicated in another study on dentists [38]. 

The evidence from this study is far from providing statistically generalizable results, as it only included thirty-four public dentists working in four cities in Turkey. Another limitation is related to the discussion of the phenomenon only from the perspective of dentists. Nevertheless, the present work is one of the few studies focusing on the experiences of HCWs appointed to missions under pandemic or similar circumstances with no prior experience or relevant training. In this regard, our findings could be used for establishment and management of appointment procedures for HCWs, while also shedding some light on more efficient planning of CICT practices. 

## Conclusions

This study identified the negative impacts of the COVID-19 pandemic on the health status, family lives and professions of the dentists assigned to CICT, illuminating serious difficulties they encountered in coping with such challenges. Our findings indicate the need for strong public policies to provide social, economic, and medical support to dentists and HCWs assigned to CICT. It is also shown that under crisis circumstances, dentists and HCWs should be empowered by all necessary training and preparations prior to their assignments. 

## Notes

### Competing interests

The authors declare that they have no competing interests.

### Authors’ ORCID

- Ozlem Ozkan: 0000-0003-1063-7489
- Neslihan Sevim : 0000-0002-3371-5331
- Zeliha Ocek: 0000-0002-5806-7035

## Supplementary Material

Themes, categories and selected quotes from interviews
