# Supplementary material for: Deployment of dentists in COVID-19 case investigation and contact tracing: An example from Turkey
Source: GMS Hyg Infect Control. 2023 Nov 29;18:Doc27. doi: 10.3205/dgkh000453 (PMC10726721; doi:10.3205/dgkh000453)
Supplement: Themes, categories and selected quotes from interviews [file HIC-18-27-s-001.pdf]

Attachment

Themes	Categories	Selected quotes
**Preparation for CICT**	Assignment	We did not know where we would be assigned, what we would do. Even the chief physician didn't know. We also didn't know what contact tracing is. Now, when I have a mission, I have no idea about the duration, location and even the content! It has been five months already but there is considerable ambiguity (Female, Izmir)
	Training	A whatsapp group was established and several videos were sent over that group. As if I have to go over all of those videos until 12 in the evening!!! I skipped some parts and tried to understand by asking colleagues (Female, Ankara)<br>We objected…You are showing us a long stick and then telling us to put this initially into the mouth deep into the area where tonsils are located, then into the nose of the patient. The doctor there said, "you know the anatomy of the head-neck very well, this is a 4 cm long stick and when you put it into mouth, it will go, so what?" (Female, Izmir)
**Basic needs**	PPE	We already normalized/got used to getting dressed at the entrance of the apartment building. When you mentioned this, I just asked myself: 'has that really been an issue for us?' (Male, Diyarbakir)<br>Making visits to several places with the same jumpsuit and spending a whole day with an infected body suit…Trying to maintain hygienie with changing gloves just once. You are doing this at 37°C, under the jumpsuit with an N95 facemask. Once you put on the facemask, you do not have any chance to take it off until the end of the mission. On the very first day, I was bewildered, unaware of what we are doing and spent the whole day without even food and water…I mean you do not feel that the pot is boiling as long as you are in it. But gradually you become experienced. (Male, Ankara)<br>I obtained and purchased softer and better fitting N95 masks myself (Male, Ankara)
	COVID-19 testing	We kindly requested it but this didn't work. How valuable is this! Everyone got tested but we haven't had any at all. At least, those who were assigned for contact tracing for one month could be tested (Female, Izmir)
	Mobile communcation	We use our mobile phones for navigation to find the places, for CICT application and for communicating with patients. Our phones have become public phones. Patients call at 11 pm with their questions. (Female, Ankara)
	Personal needs	For four months on the mission, I had no lunch at all. Drinking water and the need to use the toilet is a significant challenge. You refrain from drinking water and it is almost impossible to find a toilet. Also, how would you touch and remove your mask and then be able to drink water? (Male, Ankara)<br>There are improvements about the meals. We have the right to have a lunch break at noon, we come and have lunch at the institution and afterwards go back to the neighbourhood for the mission. (Female, Ankara)

Themes	Categories	Selected quotes
**Basic needs (continued)**	Security	Our managers called one of our female dentist colleagues at 10 pm to ask her to deliver medicine immediately to a rural household. The village was not close to the Diyarbakir city at all (Male, Diyarbakir)<br>Some citizens object to doing a nasal swab. For instance, they shout at us for pushing the stick further…Some neighbourhoods are dangerous, normally you would not pass by there. I prefer to leave those areas silently (Female, Izmir)<br>Once I was telling the patient about the cash penaly in case of noncompliance with the obligatory isolation. The patient started to shout at me by saying “what shall I do, shall I starve to death?”. Once they broke the glasses of our driver. We even called the police but police did not show up at all. (Female, Istanbul)<br>I did not encountered those…Actually I am a bit of a hardass at work. I am 1.85m tall and my colleague is around 1.80m and he is overweight too. I mean, when they see us, they somehow chicken out! (Male, Istanbul)
	Transportation	I used the navigation on my mobile, I was telling the driver the route for the address…Some of the drivers drove very fast, I warned them many times. The roads or the ramps around have already been damaged…We were about to have accident for many times, almost about to die! (Male, Ankara)<br>It is impossible without the car. Our mission sometimes finishes around 12 midnight. We also have missions to the villages in the countryside and there are no regular and proper bus services to there. Of course, you have to fill the car with fuel at your own expense (Female, Ankara)
**Work relations**	Public	There are many reactions to us. Some say “do not enter the neighbourhood with jumpsuits on, you are unveiling us”. Some say “you lock us down at home for 14 days but we have to work, who will take care of us?”. Some do not let us enter the house. There are also those who rejected male doctors. (Female, Diyarbakir)<br>They are trying to keep us in the house by asking questions non-stop even with our jumpsuits on. We try to persuade them by saying “look, we have more places to visit and staying longer in this jumpsuit is not suitable as we continuously sweat within this”. But they are not convinced, rather they say “you are not fulfilling your duty” and file complaints about us to the directorate of health (Female, Istanbul)<br>Yes, there are also some people who pray to us or say “we are so glad to have you here” (Female, Izmir)
	Managers	The managers give the impression that they always keep an eye on us. When you are on mission, you are assigned more and more patients and you are expected to visit them before you come back to the centre. For instance, once we were on a mission to a neighbourhood far from the city centre and we finshed the daily case load by 9 p.m. On our way back, we noticed that new patients had just been assigned to us from the same neighbourhood and we were expected to visit them in this same mission and to finish up with the cases before we return to the centre…We later learnt that our managers had been able to track us on the digital map to see where we are and what we are doing (Female, Ankara)<br>Our working days and working hours were changing all the time. This was not because of the changes in the case load but rather due to our manager and mostly on the basis of our attitudes towards our manager, like “carrot-and-stick”. (Female, Istanbul)<br>I finished my assignment and thanked our provincial health director. We all witnessed that he was trying to be fair (Male, Izmir)

Themes	Categories	Selected quotes
**Work relations (continued)**	Health team	We are accompanied by one health staff member only on the missions for collecting nasal swabs. Even no health staff at all for some cases. Most of the time it is only me and the driver… (Female, Istanbul)<br><br>I did not have the health staff from my clinic or the same driver as the same team members at all. Once with a health technician, once with a senior physician for a week only… The team has not been changed yet – just for three days now with the same team members! (Female, Ankara).
**Working conditions**	Working conditions	We worked very irregularly. We were estimating to finish by 5pm but most of the time we worked until 8pm. That is to say I need to finish all the visits assigned for the day. The most critical issue is that our shift does not finish at all. When you look into the official documents, it indicates the start of the shift as 10 am but the time for the end of the shift is not indicated, meaning that whenever the visits are completed. There is no concept of shift. Almost every minute a new case is registered on the system (Female, Izmir)<br><br>There is no such thing as an official break, rest time or even lucnh break during the day (Female, Istanbul)<br><br>Our leave of absence was completely cancelled by the Ministry. We had serious problems. We even did not stop on official holidays etc., we worked all the time, at the eid (eid is a religious holiday) breaks, at the weekends, during the lockdowns…That is to say, when people were staying at home, we were all working…Medical doctors do not want to give us a medical clearance for leave of absence. They refrain because they would like to avoid being the target of any official investigations …My colleague was diagnosed with cystitis and she could not get a medical clearance at all (Female, Istanbul)<br><br>Maybe I feel better about the workload because we go on missions three times in a week (Female, Istanbul)
**Being a dentist assigned to CICT**	Family life	My family still does not know that I work here….I did not want to make them worry, so I did not tell about it… (Male, Istanbul)<br><br>My wife was pregnant when we had restrictions for intra-province travels. Both of us were at home and I was really worried that she would be infected. I had difficulties with my wife on seperating our rooms at home…At my second assignment, I took my wife and my newborn daughter to stay with my mother-in-law. (Male, Izmir).<br><br>I have not seen my daughter for five months, I have been working until 12 am…Even though I am divorced, I had to give her to her father. We live in the same city, we make video calls everyday. I have not had time to complete her school registration yet. Not only there is the issue of infecting her, but also her grandmother has cancer and she had chemotherapy just before the pandemic… (crying) (Female, Diyarbakir).<br><br>There is the advantage of having a three-storey house. I have allocated the entrance flat to myself (Female, Ankara)
	Volunteering	Our previous working conditions were more severe and there were no precautions taken to protect us …Now I feel much more secure. Nevertheless, it does not matter here or there, we are caught between the devil and the deep blue sea (laughing) (Female, Ankara)<br><br>I was conscious about my responsibilities towards the society. I would prefer getting infected from the healthcare system that I am a part of rather than getting infected by some other means, for instance from the supermarket etc. I have been volunteering for this because it is my historical or personal responsibility (Male, Diyarbakir)<br><br>I aligned myself with it, I got used to it. As a matter of fact, despite such chaos, I would go if I were re-assigned. In my opinion, this is a war… (Female, Izmir)

Themes	Categories	Selected quotes
**Being a dentist assigned to CICT (continued)**	Job description and competence	We registered entries into the CICT, we took medicine to the cases, we prepared reports on those tested as positive and their contacts, we made all necessary calls…We are used for all sorts of errand. Any health officer can collect nasal swabs. I do not think that what we do is special (Male, Izmir)<br>We almost worked like courier or messenger ...We were assigned to visit the shops – as if we were municipal police officers. We were even assigned to missions at wedding parties as observers (Male, Ankara)<br>Our duties have consistently changed, for a week we only collected nasal swabs, then we did something else for the next week. Once they told us to deliver medicine, then they told us not to as it would be very risky? Why would we have to decide on the medication? It should be the decision of the doctors, family doctors. Also, they have introduced monitoring the quarantine of the cases lately as part of the process. Meaning that we are monitoring and checking whether the cases are staying at home or not. As if we had the authority to impose sanctions if they are not… (Female, Istanbul)<br>We are doing a work that is very appropriate with our job description. I mean shouldn't we do it? Who else should? We are better at collecting nasal swab than medical doctors. Pandemic is itself an extraordinary situation (Male, Istanbul)<br>As we are working at the oral area, for this reason, I do not consider this as improper (Male, Izmir)<br>I consider myself as a health professional. It is not necessary to become health professional by filling or pulling teeth (Female, Ankara)
	Payments	The State should have been honest. As everyone knows, it is evident that it is untrue to say the health workforce has become rich. They made us very high payments for three months – as if throwing us a bone, then nothing else afterwards…(Male, Izmir)<br>Everyone is working very altruistically. Ours [payment] have a surcharge but the other team members do not have this…This is inequality (Female, Istanbul)
	Dentist from MoH perspective	I am a pawn. If I die, there are many dentists to take over. This is annoying. The Ministry begrudged even thanking us. (Female, Izmir)<br>We do not need a special compliment or appreciation, it is enough as long as they acknowledge the work done.... Though what we do is not very special (Male, Ankara).<br>They should have done things to motivate us rather than applauding…They will use and just throw us away just like a wet wipe (laughing) (Male, Istanbul)<br>Such exploitation or degrading of labour for those working is too much. It is also too much to treat us as if we are robots. They do not organize decent working hours or working conditions.

Attachment to: Ozkan O, Sevim N, Ocek Z. Deployment of dentists in COVID-19 case investigation and contact tracing: An example from Turkey. GMS Hyg Infect Control. 2023;18:Doc27. DOI: 10.3205/dgkh000453

Themes	Categories	Selected quotes
**Being a dentist assigned to CICT (continued)**	Emotions	All of us are thinking about resigning. We are not allowed to have leave of absence. Many of us have been on analgesics everyday to be able to work (Female, Diyarbakir)
		One feels uncomfortable, as if the labor peace is distorted, the notion of equality is undermined, your labour is undervalued. Indeed, we are working under very unfavorable conditions so that I am almost beyond feeling… (Male, Istanbul)
		There was a call, telling me to go to the morgue downstairs. Then, I was told to collect nasal swab from the corpse…I did it. I noticed - not at that time but later - that I was very much affected by this, my anxiety level increased (Female, Izmir)
		In both of my assigments, I personally felt as if I am of no significance. There was nothing like professional satisfaction in it. When everything suddenly got normalized by June, real chaos occurred. It’s really disappointing to see everyone disregarding the rules as if everyhing is normal (Female, Istanbul)
		It was a very good experience to be a part of this work and of the team, I was happy about it but not everyone understands and feels similar. We [dentists] also have to take on responsibility. We are a part of this healthcare community…(Male, Diyarbakir)
		When I received the first notification for assignment, I felt happy because I would be beneficial to the society…Our teacher teammates brought us lunchboxes every day…there was unity among us. Each team member was sharing the work. Do I have the same feelings now? No (Female, Izmir).
**Management of the pandemic**	State	The practices of the state are entirely interesting. Like dystopia.. (Female, Ankara)
		There were measures like lockdowns. We somehow managed to take under control. When we visited the household, the patient did not go out at all. After the immediate normalization in June, everything turned into real chaos. (Female, Istanbul)
		That is to say, as long as daily life is this hectic and the workplaces, for instance textile workshops, are this crowded, positively diagnosed cases began to find it meaningless to be held under quarantine at home (Male, Istanbul)
	Ministry of Health (MoH)	The Ministry has never been transparent in announcing the official COVID-19 data. We still have no idea about the total number of infected health professionals and dentists and total number of deaths among these groups since the beginning of the pandemic. (Male, Istanbul)
		[MoH] could have used an equal, supportive and protective discourse towards all health professionals. Applauding at the balconies is cheap! There could have been several motivating interventions for us. Nasal swabs are not collected from us unless we have complaints…We are obliged to work even if we are ourselves the contacts of a patient…COVID-19 should be regarded as an occupational disease (Male, Istanbul)
		I feel uncompfortable with the managers’ attitudes, that they are only interested in the quantitative aspect of the missions, such as the number of cases visited, the number of files filled in etc. and they cannot manage to organize this contact tracing process well (Female, Ankara).
		I personally trust the Minister of Health. Everyday he shows up on TV and gives updated information. He is so sad and sleepless. I would support him only for this reason. On the other hand, I also wonder why the data lately have not been accurately announced… (Female, Izmir)
	Public	There are issues beyond the Minister; the meetings, weddings, organizations etc. all are bullshit. We were at a neighbourhood the other day for CICT and there was a crowded wedding, people gathered and were having fun. That will never do! (Female, Istanbul)
		I sent the police there once, but the people in quarantine go out anyway. One patient said: “I am willing to pay the fine whenever necessary because I have to go out”. Unless there are strict measures taken, everything is like whistling in the wind! (Female, Ankara)

Attachment to: Ozkan O, Sevim N, Ocek Z. Deployment of dentists in COVID-19 case investigation and contact tracing: An example from Turkey. GMS Hyg Infect Control. 2023;18:Doc27. DOI: 10.3205/dgkh000453
